# The role of elective neck dissection in T1 and T2 nasal cavity squamous cell carcinomas

**DOI:** 10.1007/s00405-022-07718-8

**Published:** 2022-11-07

**Authors:** Claudia Lill, Boban M. Erovic, Rudolf Seemann, Muhammad Faisal, Klaus Stelter, Bernd Gandler, Florian Frommlet, Andreas Strobl, Michael Formanek, Stefan Janik

**Affiliations:** 1 Institute of Head and Neck Diseases, Evangelical Hospital, Vienna, Austria; 2 ENT-Centre Mangfall-Inn, Rosenheim, Germany; 3 Department of Otorhinolaryngology, Head and Neck Surgery, Clinic Klagenfurt, Klagenfurt, Austria; 4 Center for Medical Statistics, Informatics, and Intelligent Systems, Medical University of Vienna, Vienna, Austria; 5 Department of Otorhinolaryngology, Head and Neck Surgery, Ordensklinikum Linz, Linz, Austria; 6 Department of Otorhinolaryngology and Phonetics, Hospital of St. John of God, Vienna, Austria; 7 Medical School, Sigmund Freud University, Vienna, Austria; 8 Department of Otorhinolaryngology, Head and Neck Surgery, Medical University of Vienna, Vienna, Austria

**Keywords:** Elective neck dissection, Nasal cavity squamous cell carcinoma, Early-stage sinonasal carcinoma, Risk score, Regional recurrence

## Abstract

**Purpose:**

To evaluate the role of elective neck dissection (END) on oncological outcome in early-stage nasal cavity squamous cell carcinomas (SCCs).

**Methods:**

In total, 87 patients with T1 (*n* = 59; 67.8%) and T2 (*n* = 28; 32.2%) SCCs were evaluated regarding performance of END, regional recurrences (RR) and its impact on cancer-specific survival (CSS). We further created a risk score based on T-classification, tumor subsite and grading to identify patients whom may benefit from END and calculated the corresponding numbers needed to treat (NNT) to prevent RR.

**Results:**

Nine (10.3%) patients experienced RR of whom 3 (5.1%) were T1 and 6 (21.4%) T2 tumors (*p* = 0.042). All RR originated from moderately or poorly differentiated (G2–G3) SCCs of the nasal septum or vestibule. END was done in 15 (17.2%) patients and none of those experienced RR (*p* = 0.121). Onset of RR represented the worst prognostic factor for CSS (HR 23.3; *p* = 0.007) with a 5y-CSS of 44.4% vs. 97.3% (*p* < 0.001). RR occurred in none of the patients with no or low risk scores compared to 31.6% (6/19) in patients with high-risk scores (*p* = 0.006). Accordingly, three high-risk patients would need to undergo END (NNT 2.63) to prevent RR compared to a NNT of 8 for the whole cohort.

**Conclusions:**

Although rare, occurrence of RR significantly deteriorates outcome in early stage nasal cavity SCCs, which could be effectively reduced by performance of END. The importance of END is currently underestimated and our proposed risk score helps identifying those patients who will benefit from END.

## Introduction

Sinonasal squamous cell carcinomas (SCCs) account for less than 3% of malignant tumors of the upper aerodigestive tract and for 3% of all head and neck carcinomas [1, 2]. The American Joint Committee of Cancer (AJCC) differentiates between sinonasal carcinomas originating either from the (i) nasal cavity and ethmoidal sinus or the (ii) maxillary sinus [3]. The majority of SCCs are located in the nasal cavity (43.9–45.7%) followed by the maxillary sinus (33.3–35.9%) with nodal involvement in 14.2% of cases (range 4–27%) [2, 4, 5].

Primary tumor resection with free resection margins followed by adjuvant radiotherapy (RT) in selected cases represents the mainstay of therapy [6, 7]. Elective neck treatment is considered only in high-risk tumors or advanced T-classifications [8]. Regional recurrences (RR) are found in 18.1% of nasal cavity SCCs, which can be reduced by elective nodal treatment to a 4% rate [2, 8]. Although complications are rare for experienced head and neck surgeons, they naturally occur also after elective neck dissection (END). Shoulder immobility or spinal accessory nerve palsy is reported as the most common complication occurring in 10% of patients after selective or modified radical neck dissection [9], but with preservation of normal shoulder function in 93.8% of cases [10].

However, although the risk of RR can be indeed reduced by incorporating elective nodal treatment [2, 8, 11], END failed to demonstrate significantly better overall survival in T3–T4 sinonasal SCCs [12], while elective neck treatment was associated with better prognosis in higher stage maxillary sinus SCCs [13–15]. Hence, elective nodal treatment is currently not recommended for stage I and II sinonasal SCCs, as its benefit is still under debate [16].

To shed some light on this matter, we performed this retrospective, multicentric analysis of 87 patients with stage I and II nasal cavity SCCs. Since our working group has demonstrated differences regarding clinical behavior of nasal cavity SCCs based on anatomic subsites [17], we were particularly interested in the risk of RR based on anatomic subsites. Moreover, we identified risk factors for RR that were further used for creation of a risk score that proved to predict oncological outcome.

## Materials and methods

### Study cohort

A retrospective, multicenter chart review of 87 patients with cT1N0 (*n* = 58; 66.7%) and cT2N0 (*n* = 29; 33.3%) nasal cavity SCCs was performed. Tumors originating from paranasal sinuses (e.g., maxillary sinus or ethmoidal cells), T3–T4 tumors, cases with lymph node involvement and other histologies than SCCs were excluded. Data of patients were provided by attending centers and evaluated individually regarding appropriateness by two authors (CL, SJ). All patients were treated between 01/95 and 03/21 and the mean follow-up time was 40.5 ± 41.5 months (range 0.1–299.5 months).

### Clinical data

Clinical and sociodemographic characteristics for each patient were obtained from medical hospital records, surgical and pathological reports, and imaging findings. We were especially interested in tumor origin (tumor subsite), T-classification (T1 vs. T2), grading (G1 vs. G2 vs. G3), therapy, performance of elective neck treatment, occurrence of recurrence (local vs. regional vs. distant), and oncological outcome parameters. The decision whether to perform an END as well as its extent (level of dissection and laterality) was individually made by treating surgeons. According to the AJCC 8th edition, we differentiated tumors originating from nasal septum, nasal floor, nasal lateral wall, nasal vestibule or nasal cavity not otherwise specified [3].

### Oncological outcomes

We used the cancer-specific survival (CSS), occurrence of local (LR) or regional recurrence (RR) and the freedom from regional recurrence (FFRR) as oncological outcome parameters. CSS was calculated from date of surgery to date of death from sinonasal carcinoma, while FFRR was calculated only in patients who were assumed to be “free of cancer” from date of surgery to date of RR.

### Statistical methods

Statistical analysis was performed using the SPSS software (version 27; IBM SPSS Inc., Chicago, IL, USA). Data are indicated as absolute numbers with corresponding percentages in brackets. The Chi-square test was used to assess associations between nominal variables. An unpaired student’s *T* Test was used to compare means of normally distributed variables. Univariable cox-regression analysis was performed to evaluate the impact of different clinical variables on FFRR and CSS. Kaplan–Meier analysis and Log-rank test were assessed for survival analysis. A binary logistic regression analysis, in turn, was applied to screen clinical variables regarding their potential for predicting RR. ROC (receiver operating characteristic) analyses were subsequently performed to quantify this predictive power and corresponding areas under the curve (AUC) are indicated. In addition, we calculated the number needed to treat (NNT) for END to prevent RR. All tests were two- sided, and *p* values below 0.05 were considered statistically significant.

## Results

### Patient cohort

In total, our patient cohort consisted of 87 patients, 37 females (42.5%) and 50 males (57.5%), with a mean patient age of 60.6 years (range: 33.6–89.2 years). Tumors most commonly originated from the nasal vestibule (*n* = 40; 46.0%), followed by the nasal septum (*n* = 29; 33.3%) and the lateral nasal wall (*n* = 18; 20.7%; Table 1). None of the included cases originated from the nasal floor nor from the nasal cavity non otherwise specified. All septal SCCs were located in the cartilaginous anterior part of the septum. We had 59 (67.8%) T1 and 28 (32.2%) T2 tumors and all patients presented clinically with cN0 necks. The majority of nasal cavity SCCs (*n* = 53; 60.9%) showed moderate-differentiation (G2), which was neither affected by tumor origin (*p* = 0.327), age (*p* = 0.442) nor T-classification (*p* = 0.563).

**Table 1:** Study cohort and type of recurrence

Variables	Total	Type of recurrence			*p* value
		Local	Regional	No	
	*n* (%)	*n* (%)	*n* (%)	*n* (%)	
Sex					
Male	50 (57.5)	8 (16.0)	4 (8.0)	38 (76.0)	
Female	37 (42.5)	4 (10.8)	5 (13.5)	28 (75.7)	0.594^a^
Tumor site					
Septum	29 (33.3)	4 (13.8)	5 (17.2)	20 (69.0)	
Lateral wall	18 (20.7)	3 (16.7)	0 (0)	15 (83.3)	
Vestibule	40 (46.0)	5 (12.5)	4 (10.5)	31 (77.5)	0.450^a^
T-classification					
T1	59 (67.8)	10 (16.9)	3 (5.1)	46 (78.0)	
T2	28 (32.2)	2 (7.1)	6 (21.4)	20 (71.4)	**0.042**^a^
Grading					
G1	12 (13.8)	3 (25.0)	0 (0)	9 (75.0)	
G2	53 (60.9)	7 (13.2)	6 (11.3)	40 (75.5)	
G3	22 (25.3)	2 (9.1)	3 (13.6)	17 (77.3)	0.560^a^
END					
Yes	15 (17.2)	1 (6.7)	0 (0)	14 (93.3)	
No	72 (82.8)	11 (15.3)	9 (12.5)	52 (72.2)	0.195^a^
Adjuvant therapy					
Yes	14 (16.1)	1 (7.1)	1 (7.1)	12 (85.7)	
No	73 (83.9)	11 (15.1)	8 (11.0)	54 (74.0)	0.633^a^

Data of patients regarding sex, tumor site, T-classification, grading, elective neck dissection (END) and adjuvant therapy are indicated according to occurrence and type of recurrence. No distant recurrences have been detected. Absolute numbers (*n*) with corresponding percentages are indicated within brackets

Bold inidcate *p* values below 0.05 were considered as statistically significant

^a^Chi-square test

### Therapy

Surgical tumor resection was applied in all patients and ranged from partial lateral rhinectomy over endoscopic resections to total rhinectomies*.* Free resection margins (R0) were achieved in 82 (94.3%) of those. Surgery alone was performed in 72 patients (82.8%), surgery and adjuvant RT in 14 (16.1%) and one patient (1.1%) received trimodal therapy consisting of surgery and chemoradiotherapy (CRT). Only tumor sites were irradiated either in an adjuvant or curative setting, while elective neck irradiation was not performed. Adjuvant RT was applied significantly more often in patients with incomplete tumor resections (80% vs. 12.2%; *p* = 0.002) and in T2 tumors (32.1% vs. 8.5%; *p* = 0.010). An END was performed in 15 patients (17.2%), whose characteristics are indicated in Table 2. One occult neck node metastasis (1 out of 12 resected lymph nodes) was found in one electively neck dissected patient (1.1%).

**Table 2:** Elective neck-dissection

Case	Sex	Age	Tumor	Tumor site	TNM	Grading	Risk score	END	Level
1	M	36y	Primary	Septum	T1 N0	G2	Moderate	Bilateral	1–3
2	M	69y	Primary	Septum	T1 N0	G3	Moderate	Ipsilateral	1–2
3	M	73y	Primary	Vestibule	T2 N0	G2	High	Bilateral	1–3
4	M	61y	Primary	Vestibule	T2 N0	G1	Moderate	Bilateral	1–2
5	F	44y	Primary	Vestibule	T1 N0	G2	Moderate	Bilateral	1–2
6	M	62y	Primary	Vestibule	T1 N0	G2	Moderate	Ipsilateral	1–2
7	F	58y	Primary	Lateral Wall	T2 N0	G2	Moderate	Bilateral	1–3
8	M	49y	Recurrence	Lateral Wall	T2 N0	G2	Moderate	Bilateral	1–2
9	M	59y	Primary	Lateral Wall	T1 N0	G2	Low	Ipsilateral	1–3
10	M	59y	Primary	Lateral Wall	T1 N0	G3	Low	Bilateral	1–3
11	M	66y	Primary	Vestibule	T1 N0	G2	Moderate	Ipsilateral	2–4
12	F	71y	Primary	Vestibule	T2 N0	G2	High	Ipsilateral	2–3
13	M	47y	Primary	Vestibule	T1N0	G2	Moderate	Ipsilateral	1–2
14	M	54y	Primary	Vestibule	T2N1	G1	Moderate	Ipsilateral	1–3
15	F	65	Primary	Septum	T2N0	G3	High	Bilateral	1–3

Demographics of patients with stage I and II squamous cell carcinomas of the nasal cavity undergoing ipsilateral or bilateral elective neck dissection (END)

### Recurrence

Recurrences occurred in 21 patients (24.1%) comprising 12 local (13.8%) and 9 regional (10.3%), but no distant failures. The overall mean and median time between diagnosis and recurrence was 35.0 and 13.5 months, respectively. Noteworthy, RR occurred two times earlier compared to LR (22.5 vs. 44.3 months), more often in T2 tumors (20.7% vs. 5.2%; *p* = 0.055) and in cases with positive lymphovascular invasion (LVI; *p* = 0.011). Regarding to resection margins, RR occurred in one patient after incomplete tumor resection compared to 8 in those with free resections margins, which was higher but not statistically significant (20.0% vs. 9.8%; *p* = 0.670). The FFRR was also not significantly affected by incomplete tumor resection (*p* = 0.941). RR did not occur in any patient with lateral nasal wall tumor, but in 17.2% of septal and 10% of nasal vestibule carcinomas (Table 1). Consequently, the 5y-FFRR was 83.6% in tumors of the nasal vestibule or septum compared to 100% in lateral nasal wall tumors (*p* = 0.090; Table 3). Only T2 carcinomas represented a significant worse prognostic factor for development of RR (HR 4.03; *p* = 0.048; Table 3). In turn, performance of END did not represent an overall prognosticator for FFRR (HR 0.03; *p* = 0.342). Yet, none of the 15 electively neck-dissected patients experienced RR (*p* = 0.121). Similarly, no RR was observed in well-differentiated (G1) tumors compared to 11.3% in moderately differentiated (G2) and 13.6% in poorly differentiated (G3) tumors (*p* = 0.428).

**Table 3:** Freedom from regional recurrence

Freedom from regional recurrence							
Variables	Log-rank test			*p*	Cox-regression analysis		
	1 y	3 y	5 y		HR	*p*	95% CI
Sex							
Male	95.1	88.3	88.3		1.55	0.517	0.41 to 5.86
Female	89.4	85.4	85.4	0.514	1		
Age							
< 62 y	97.6	91.5	91.5		0.37	0.159	0.10 to 1.48
≥ 62 y	87.1	81.7	81.7	0.142	1		
T-classification							
T1	97.4	94.6	94.6		1	**0.048**	1.01 to 16.2
T2	84.1	73.8	73.8	**0.032**	4.03		
Tumor site							
Septum + vestibule	90.7	83.6	83.6		31.3	0.302	0.05 to> 100.0
Lateral nasal wall	100.0	100.0	100.0	0.090	1		
Grading							
G1	100.0	100.0	100.0		0.04	0.470	0.00 to 251.2
G2 + G3	91.7	85.2	85.2	0.256	1		
END							
Yes	100.0	100.0	100.0		1	0.342	0.03 to > 100.0
No	90.9	83.4	83.4	0.121	30.6		
Adjuvant therapy							
Yes	100.0	90.9	90.9		1	0.342	0.32 to 26.5
No	91.3	87.0	87.0	0.326	2.92		
Risk score							
High	75.0	55.6	55.6		13.9	**0.001**	2.77 to 66.7
No/low/moderate	97.9	95.6	95.6	** < 0.001**	1		

*END* elective neck dissection, *HR* hazard ratio, *95% CI* 95% confidence interval

Bold inidcate *p* values below 0.05 were considered as statistically significant

### Risk score for regional recurrence

**Fig. 1:**
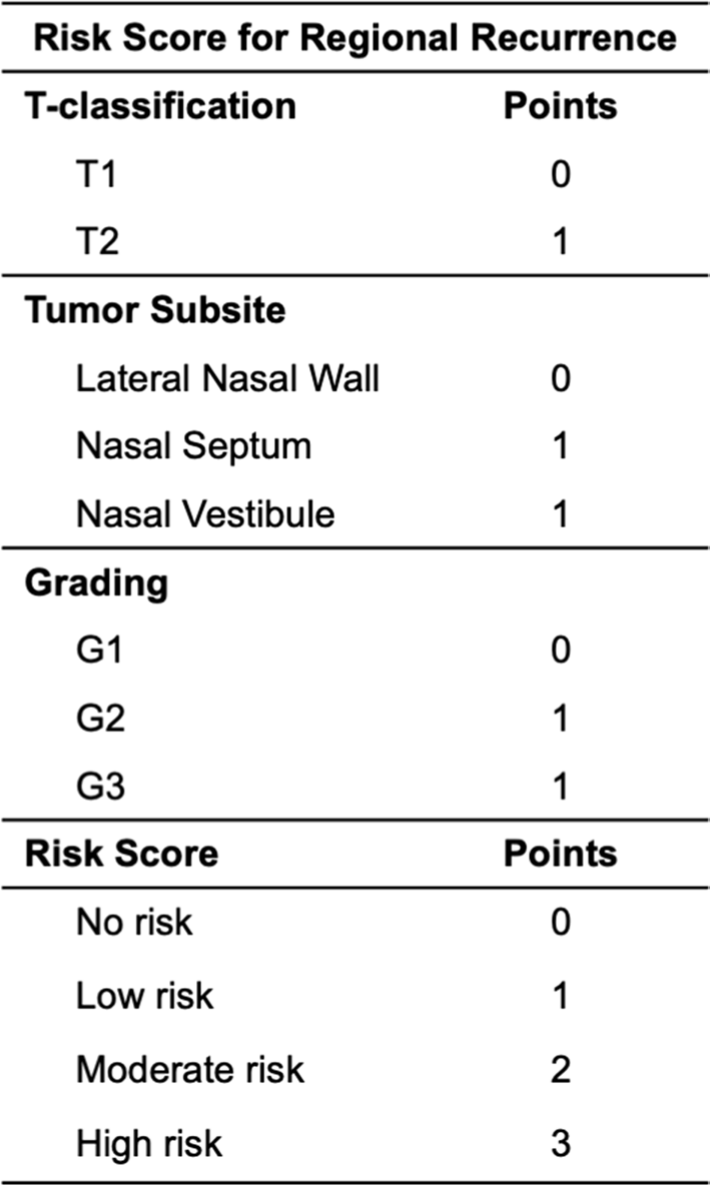
Risk score for regional recurrence

**Fig. 2:**
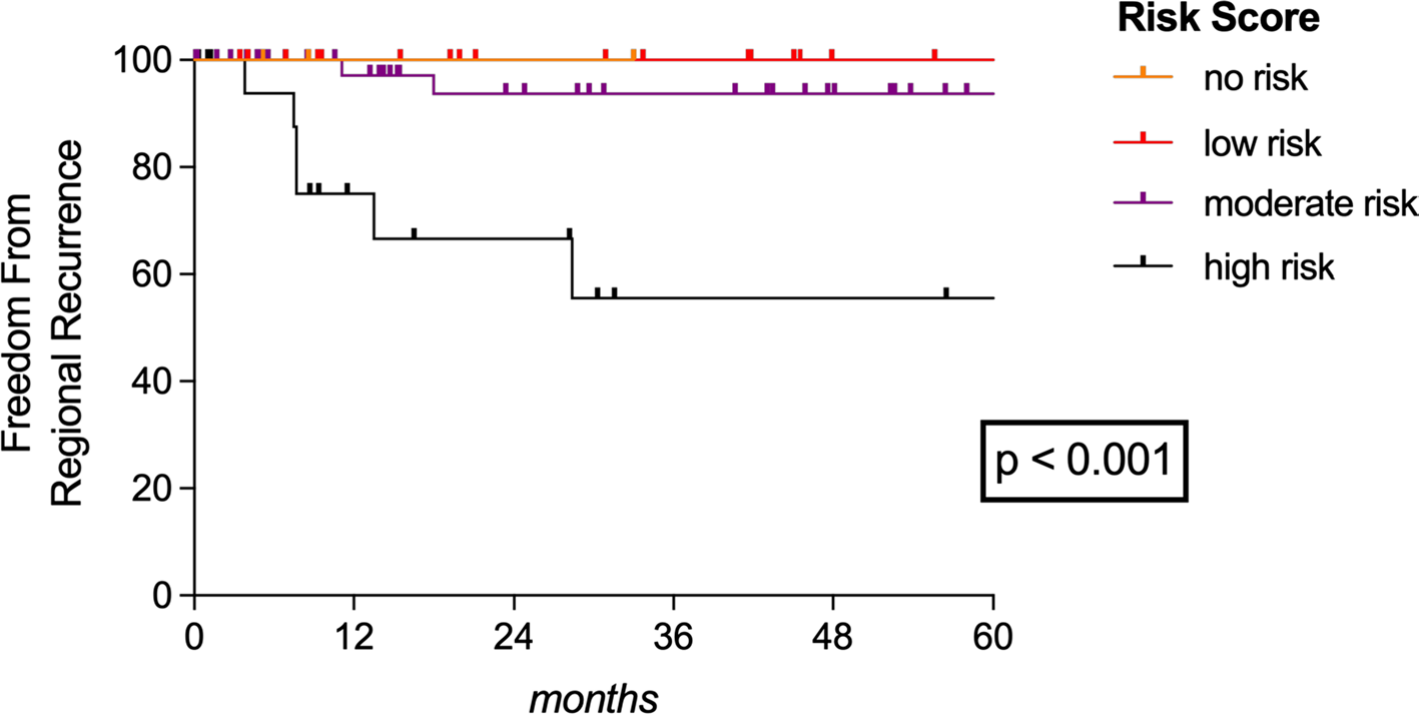
**Freedom from regional recurrence.** Freedom from regional recurrence (FFRR) plotted according to our proposed risk score differentiating between patients with no risk factors and those with low-, moderate- or high-risk scores, respectively

The AUC was 0.686 (*p* = 0.069), 0.596 (*p* = 0.340), and 0.625 (*p* = 0.259) for T-classification, grading and tumor site for predicting RR. Importantly, the highest AUC of 0.793 (*p* = 0.004) for predicting RR was found when combining T-classification (T2 > T1), tumor site (nasal septum and nasal vestibule > other subsites) and grading (G2–G3 > G1). Therefore, we set up a simple risk score based on those three variables to better predict the risk for RR (Fig. 1). As illustrated, each variable was rated with either 0 or 1 resulting in patients with no risk (0 points), low risk (1 point), moderate risk (2 points) or high risk (3 points) for RR. Applying our risk score, we had 3 patients (3.4%) with no risk, 17 (19.5%) with low, 48 (55.2%) with moderate and 19 (21.8%) with high risk for RR. No regional failures were noticed in patients with no (0/3) or low risk (0/17) scores compared to 6.3% (3/48) and 31.6% (6/19) in those with moderate- or high-risk scores (*p* = 0.006), respectively. A high-risk score was particularly associated with a 13.9-times higher risk for RR (*p* = 0.001) and the FFRR in accordance with our risk score is plotted in Fig. 2.

### Outcome analysis

After we proved that our risk score represented a poor prognosticator for RR, we were further interested in whether occurrence of RR, performance of END or our created risk score may affect CSS as well (Table 4). The CSS was significantly worse in T2 tumors (*p* = 0.003), patients with high-risk scores (*p* < 0.001) and those who experienced RR (< 0.001). In particular, high-risk scores (HR 16.0; *p* = 0.014) and occurrence of RR (HR 23.3; *p* = 0.007) was associated with a 17- and 23-fold increase risk for cancer-related death. In contrast, CSS was neither affected by any other tested variables, such as grading (*p* = 0.342), resection margins (*p* = 0.283), sex (*p* = 0.434), age (*p* = 0.217) or performance of adjuvant therapy (*p* = 0.827).

**Table 4:** Cancer-specific survival

Cancer-specific survival							
Variables	Log-rank test			*p*	Cox-regression analysis		
	1y	3y	5y		HR	*p*	95% CI
Sex							
Male	100.0	96.4	88.0		1		
Female	100.0	100.0	92.3	0.434	0.42	0.448	0.04 to 4.01
Age							
< 62 y	100.0	96.3	96.3		1	0.251	0.39 to 35.7
≥ 62 y	100.0	100.0	82.7	0.217	3.77		
T-classification							
T1	100.0	100.0	100.0		1	0.274	0.01 to > 1000
T2	100.0	94.1	70.8	**0.003**	200		
Tumor site							
Septum + vestibule	100.0	97.4	90.9		1	0.693	0.16 to 15.3
Lateral nasal wall	100.0	100.0	87.5	0.691	1.58		
Grading							
G1	100.0	100.0	100.0		0.04	0.552	0.0 to > 1000
G2 + G3	100.0	97.5	88.1	0.342	1		
END							
Yes	100.0	100.0	100.0		1		
No	100.0	97.4	86.4	0.205	34.2	0.450	0.00 to 250.0
Adjuvant therapy							
Yes	100.0	100.0	87.5		1		
No	100.0	97.4	90.5	0.827	0.78	0.827	0.08 to 7.48
Risk score							
High	100.0	88.9	55.6		16.9	**0.014**	1.76 to 166.7
No/low/moderate	100.0	100.0	97.2	** < 0.001**	1		
Regional recurrence							
Yes	100.0	83.3	44.4		23.3	**0.007**	2.39 to 200.0
No	100.0	100.0	97.3	** < 0.001**	1		

*END* elective neck dissection, *HR* hazard ratio, *95% CI* 95% confidence interval

Bold inidcate *p* values below 0.05 were considered as statistically significant

The 5-year CSS was almost halved in patients with high-risk scores (55.6% vs. 97.2%; *p* < 0.001), and more importantly, 55.6% of patients experiencing RR died from cancer within the first 5 years after initial surgery (*p* < 0.001). Once more, none of the END patients died from cancer-related reasons during the first 5 years after surgery.

### Number needed to treat

**Fig. 3:**
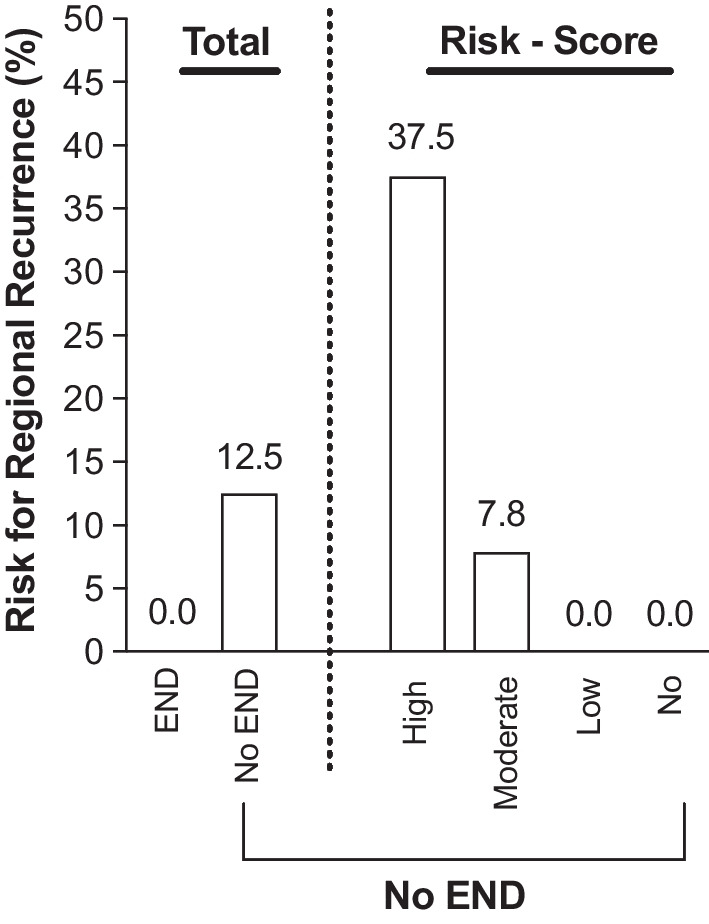
**Risk for regional recurrence.** The risk for occurrence of a regional recurrence (RR) is indicated according to our proposed risk score and performance of elective neck dissection (END)

Occurrence of RR poses the worst prognosticator for CSS, which occurred in 10.3% of cases. Performance of END proved to effectively reduce the risk for RR (*p* = 0.121) with an overall NNT of 8 to prevent RR. By applying our risk score, and the NNT was 2.63 for high-risk patients compared to a NNT of 12.5 in those with a moderate-risk score (Table 5, Fig. 3).

**Table 5:** Number needed to treat to prevent regional recurrence

	Total		Risk score							
			High		Moderate		Low		No	
	Yes	No	Yes	No	Yes	No	Yes	No	Yes	No
END	15	72	3	16	10	38	2	15	0	3
Occurrence of RR	0	9	0	6	0	3	0	0	0	0
Risk for RR	0	0.125	0	0.38	0	0.08	0	0	0	0
NNT	**8**		**2.63**		**12.5**		**–**		**–**	

The number needed to treat (NNT) was eight for our overall cohort to prevent the risk for regional recurrence (RR). Conversely, three patients of the high-risk cohort would need to undergo elective neck dissection (END) to prevent RR

Bold inidcate *p* values below 0.05 were considered as statistically significant

## Discussion

Adequate data regarding occult metastasis in early stage sinonasal carcinomas do not exist so far. Yet, occult metastases are reported in 12.7% of T3–T4 sinonasal SCCs [12] and 13.5–22.2% of maxillary sinus malignancies [13]. The rate of occult metastasis is indeed considered to be low in early stage sinonasal malignancies, and thereby, elective neck treatment is currently not recommended, as its beneficial effects are not thought to overweigh any treatment associated side-effects.

However, our analysis reveals that onset of RR significantly deteriorates oncological outcome and particularly T-classification represented a significantly worse prognosticator for RR. Patients with T2 tumors had a fourfold increased risk for RR and a 68.1% 5-year CSS compared to 100% in T1 tumors. Similarly, Ahn et al. reported of a remarkable higher risk for lymph node involvement in T2 (9.8%) and T3 (10.3%) nasal cavity SCCs compared to T1 (4%) tumors [2]. These data suggest that oncological behavior of T2 tumors, indicated by lymph node involvement and risk for RR, is more likely that of T3 than that of T1 tumors, respectively. Consequently, it is, therefore, essential to reconsider T2 nasal cavity tumors as more aggressive than T1 tumors that may require also adapted treatment regimes.

In our cohort, T-classification represented by far the worst prognosticator for RR followed by tumor origin and grading. Importantly, combining all three factors provided the highest AUC for predicting RR. The relevance of the T-classification on outcome of sinonasal carcinomas has already been shown [2]. Specifically, Fornelli et al. showed that SCC of the anterior nasal cavity with involvement of two or more nasal subregions corresponding to at least T2 tumors significantly shortened survival [18]. Moreover, higher tumor grading has also been linked to worse outcome [19], while there are few data evaluating the significance of tumor subunits on the outcome of SCC in the nasal cavity [17]. Indeed, RR exceptionally occurred in tumors originating from the nasal vestibule and septum with moderate (G2) or poor differentiation (G3), but not from tumors of the lateral nasal wall or well-differentiated ones (G1). According to our risk score, patients with high-risk scores showed a 14-times higher risk for RR and an almost 17-times higher risk to pass away from cancer-related causes. Although our risk score needs to be interpreted of course with some caution in the absence of application and evaluation to a control cohort, it nevertheless proves to predict the risk for RR ranging from 0% in patients with no or low risk scores to 6.5% in those with moderate scores up to 31.6% in cases with high scores.

An estimated risk for occult lymph node metastases of 15–20% is widely accepted as threshold to justify END [2, 8, 11]. Or the other way round, an NNT of 5–6 patients is currently considered as appropriate to detect one patient with occult neck node metastasis. As abovementioned, the rate of occult lymph node metastasis is considered as being low for T1 and T2 sinonasal malignancies and occult neck node metastasis were found in only one single patient (1.1%). Therefore, neither serious data on END nor on occult lymph node metastasis are available so far. Whether onset of RR in almost one-third of our high-risk patients is caused by new spread of tumor cells to initially unaffected lymph nodes or pop up of occult lymph node metastasis is unclear and needs further evaluation. Nonetheless, no RR were found in those patients who received END, which is in alignment to several studies reporting of significantly better regional control and decreased RR after elective neck treatment [12–14, 18, 20, 21].

Although complications are rare in experienced hands [9, 10], the low risk of RR or occult lymph node metastasis does not justify END in all stage I–II nasal cavity SCCs. The overall NNT of our cohort was 8, which indicates that 8 patients need END to prevent one RR. By applying our risk score, the NNT could be reduced to 2–3 in high-risk patients compared to 12.5 in patients at moderate risk. Thus, our risk score provides a useful decision tool for identifying patients in whom END should definitely be considered.

Moreover, we believe that END should be favored over elective neck irradiation in patients with higher estimated risks for RR due to following reasons: (i) still low complication rates and morbidity associated with END [9, 10]; (ii) END provides additional beneficial histopathological information (occult lymph node metastasis) resulting in accurate staging and also guides the future possibility of adjuvant chemotherapy; (iii) avoidance of radiation induced side-effects, such as xerostomia or mucositis [22]; and (iv) and irradiation could be preserved as curative treatment option in the case of neck failure, which might be severely limited by possible long-term complications such as spinal cord toxicity if irradiation was applied electively before.

The homogeneity and size of our cohort including 87 patients with T1 and T2 nasal cavity SCCs, the set-up of a risk score for regional failure and the calculation of the NNT represent the strengths of the current study. Once more, it is important to emphasize that our risk score needs to be validated with a larger patient cohort and may require further adjustment. In turn, we see three limiting factors. First, it is indeed challenging to clearly define tumors´ arising within the nasal cavity, and therefore, comparison of tumour subsites may be hampered. Second, the decreased quantity of performed ENDs as well as the individual (non-randomized) surgeon’s decision on how extensive (level, laterality) the END should be performed. And third, the retrospective study-design including a low number of patients with limited events and partially short follow-up times represent further flaws. Nonetheless, considering the current literature regarding T1 and T2 sinonasal SCCs with patient cohorts ranging between 10 and 35 proves that our cohort is representative [6, 9, 11, 17, 20, 23, 24].

## Conclusion

Occult neck node metastasis and RR are rare in early stage nasal cavity SCCs. However, occurrence of RR represented a poor prognosticator for oncological outcome, which could be effectively reduced by performance of END. Our proposed risk score helps identifying patients at higher risk for RR who may benefit from END.

## Data Availability

The data sets used and/or analyzed during the current study are available from the corresponding author on reasonable request.
